# Atrial Fibrillation Mechanisms and Implications for Catheter Ablation

**DOI:** 10.3389/fphys.2018.01458

**Published:** 2018-10-17

**Authors:** Ghassen Cheniti, Konstantinos Vlachos, Thomas Pambrun, Darren Hooks, Antonio Frontera, Masateru Takigawa, Felix Bourier, Takeshi Kitamura, Anna Lam, Claire Martin, Carole Dumas-Pommier, Stephane Puyo, Xavier Pillois, Josselin Duchateau, Nicolas Klotz, Arnaud Denis, Nicolas Derval, Pierre Jais, Hubert Cochet, Meleze Hocini, Michel Haissaguerre, Frederic Sacher

**Affiliations:** ^1^Cardiac Electrophysiology Department, Hopital Haut Leveque, Bordeaux, France; ^2^Electrophysiology and Heart Modeling Institute (LIRYC), Bordeaux University, Pessac, France; ^3^Cardiology Department, Hopital Sahloul, Universite de Sousse, Sousse, Tunisia; ^4^Cardiology Department, Wellington Hospital, Wellington, New Zealand; ^5^Department of Cardiovascular Imaging, Hopital Haut Leveque, Bordeaux, France

**Keywords:** atrial fibrillation, reentrant drivers, catheter ablation, fibrosis, mapping, pulmonary vein ablation

## Abstract

AF is a heterogeneous rhythm disorder that is related to a wide spectrum of etiologies and has broad clinical presentations. Mechanisms underlying AF are complex and remain incompletely understood despite extensive research. They associate interactions between triggers, substrate and modulators including ionic and anatomic remodeling, genetic predisposition and neuro-humoral contributors. The pulmonary veins play a key role in the pathogenesis of AF and their isolation is associated to high rates of AF freedom in patients with paroxysmal AF. However, ablation of persistent AF remains less effective, mainly limited by the difficulty to identify the sources sustaining AF. Many theories were advanced to explain the perpetuation of this form of AF, ranging from a single localized focal and reentrant source to diffuse bi-atrial multiple wavelets. Translating these mechanisms to the clinical practice remains challenging and limited by the spatio-temporal resolution of the mapping techniques. AF is driven by focal or reentrant activities that are initially clustered in a relatively limited atrial surface then disseminate everywhere in both atria. Evidence for structural remodeling, mainly represented by atrial fibrosis suggests that reentrant activities using anatomical substrate are the key mechanism sustaining AF. These reentries can be endocardial, epicardial, and intramural which makes them less accessible for mapping and for ablation. Subsequently, early interventions before irreversible remodeling are of major importance. Circumferential pulmonary vein isolation remains the cornerstone of the treatment of AF, regardless of the AF form and of the AF duration. No ablation strategy consistently demonstrated superiority to pulmonary vein isolation in preventing long term recurrences of atrial arrhythmias. Further research that allows accurate identification of the mechanisms underlying AF and efficient ablation should improve the results of PsAF ablation.

## Introduction

Atrial fibrillation (AF) is the most common cardiac arrhythmia. It represents a major cause of mortality and morbidity, mainly related to embolic events and heart failure (Benjamin et al., 1998; Ruigomez et al., 2002, 2009; Pedersen et al., 2006; Miyasaka et al., 2007; Potpara et al., 2013; Pandey et al., 2017; Eggimann et al., 2018; Reddy et al., 2018). AF is a heterogeneous rhythm disorder that is related to a wide spectrum of etiologies and has broad clinical presentations. Despite extensive research, the mechanisms underlying AF remain incompletely understood. AF results from interactions between triggers, responsible for its initiation, and the substrate responsible for its perpetuation. In addition, ionic and anatomic remodeling, genetic predisposition, and neuro-humoral contributors make these interactions more complex.

The pulmonary veins play a key role in the pathogenesis of AF and their isolation is associated to high rates of AF freedom in patients with paroxysmal AF (PAF). However, ablation of persistent AF (PsAF) remains less effective, mainly limited by the difficulty to identify the sources sustaining AF outside the pulmonary veins.

We aimed to review the mechanisms underlying AF and their implications for catheter ablation.

## AF pathophysiology

**Figure 1:**
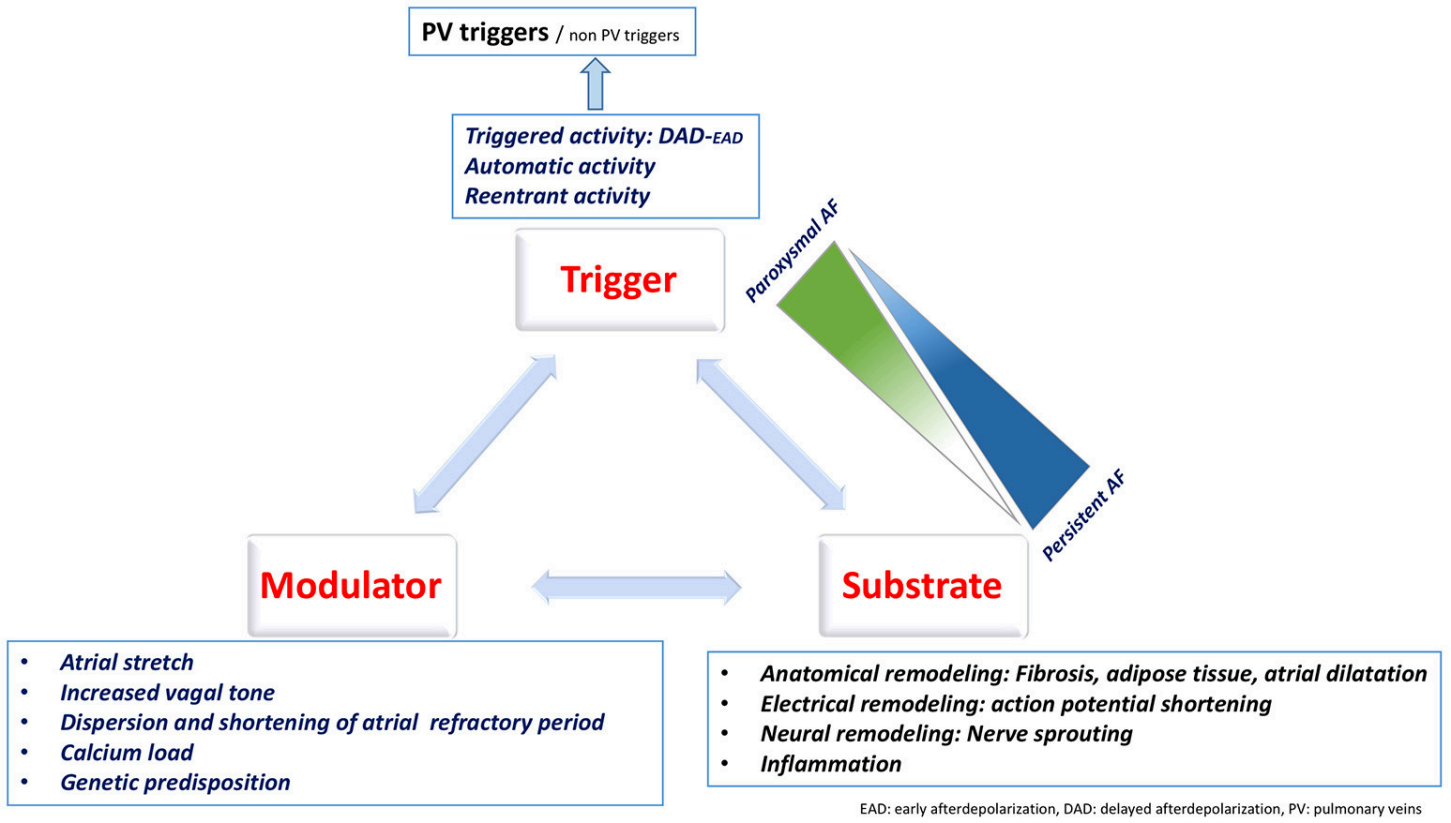
Coumel triangle summarizing the different contributors to AF.

The mechanisms underlying AF are classically described as mechanisms responsible for its initiation (triggers) and mechanisms responsible for its perpetuation (Figure 1). This classification is clinically relevant as it allows to identify therapeutic targets.

### AF triggers

Haïssaguerre et al. (1998) first reported the essential role of the pulmonary veins (PVs) in the initiation and maintenance of paroxysmal AF (PAF). Ectopic activities originating from the PVs were identified in 94% of patients suffering from frequent pre-procedural AF episodes. Discrete ablation targeting the site of origin of the ectopic activities abolished the arrhythmia and prevented its recurrence in 62% of the cases after a follow-up of 8 months. The role of the PVs in triggering AF was confirmed in multiple subsequent studies (Chen et al., 1999; Wu et al., 2001; Sanders et al., 2002; Mahida et al., 2015).

Compared to the atrial cells, the PVs cardiomyocytes have specific action potential properties that predispose to arrhythmogenesis. In fact, the PVs cells have a higher resting membrane potential, a lower amplitude of the action potential, a smaller maximum phase 0 upstroke velocity and a shorter action potential duration (APD). Slow and rapid delayed rectifier currents are greater in the PVs whereas transient outward K+ current and L-type Ca2+ current are smaller (Ehrlich et al., 2003).

The initial premature beats arising from the PVs are focal (Arentz et al., 2007). These beats are likely automatic or triggered and related to calcium handling abnormalities and subsequent delayed afterdepolarizations (DAD) (Hirose and Laurita, 2007; Takahara et al., 2011; Heijman et al., 2014). They are modulated by acute stressors like atrial stretch (Kalifa et al., 2003) and neural activation (Patterson et al., 2005; Lu et al., 2009). Early after depolarization due to prolonged action potential duration are mainly described in models of long QT syndrome (Lemoine et al., 2011). Subsequent firing is either related to DAD due to abnormal diastolic spontaneous calcium release (Chou et al., 2005; Nattel and Dobrev, 2016) or to reentrant activity at the junction between the PVs and left atrium. In fact, atrial myocytes at the entrance of the PV have abrupt changes in their fiber orientation, leading to slow conduction and reentry (Hocini et al., 2002). In addition, ionic mechanisms facilitate the occurrence of reentry by shortening the APD (increased rapid [I_Kr_] and slow [I_Ks_] delayed rectifier K+ currents) and slowing the conduction via the inactivation of Na+ currents (Nattel and Dobrev, 2016).

Additional ectopic sources triggering AF were identified outside the PVs. They can be located in the vena cavae, the crista terminalis, the coronary sinus, the ligament of Marshall, the inter-atrial septum, the appendages… (Mansour et al., 2002; Lin et al., 2003; Shah et al., 2003; Lee et al., 2005; Yamada et al., 2006; Pastor et al., 2007; Hwang and Chen, 2009; Yamaguchi et al., 2010; Elayi et al., 2013; Enriquez et al., 2017). Lin et al. (2003) identified non-PV triggers in 20% of the ectopic beats initiating PAF. Non PV triggers were predicted by female gender (odds ratio 2.00, 95% confidence interval 1.02–3.92) and left atrial enlargement (odds ratio 2.34, 95% confidence interval 1.27–4.32) in patients with PAF (Lee et al., 2005) and are more prevalent in AF of longer duration (Hung et al., 2017). Mechanisms underlying extra-PV triggers are less well-elucidated.

### Perpetuation of AF

**Figure 2:**
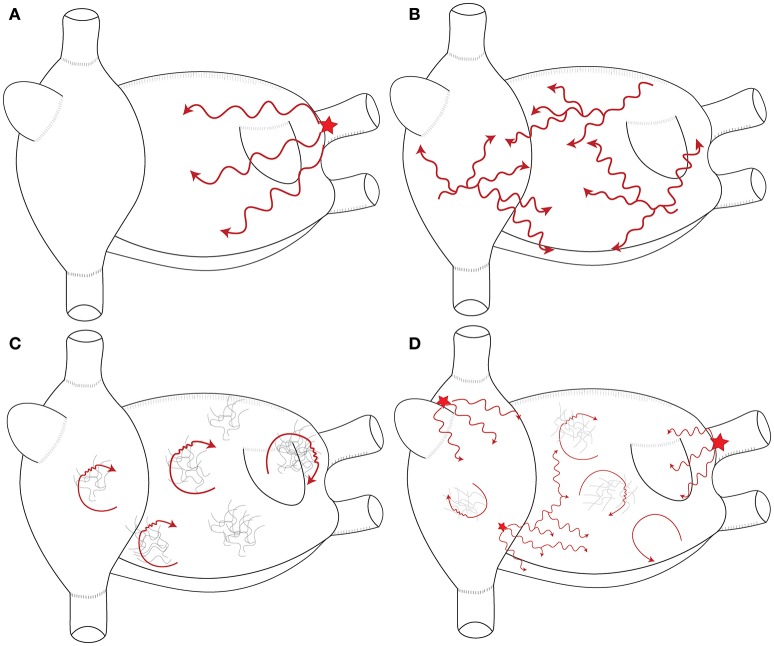
Schematic representation of the mechanisms maintaining AF. **(A)** Single stable focal or reentrant source (star) with fibrillatory conduction. **(B)** Multiple wavelets: multiple waves propagate randomly and give birth to new daughter wavelets. **(C)** Multiple reentries (red arrows) around areas of scar and fibrosis. **(D)** Combination of the different mechanisms that sustain AF in humans. These mechanisms are typically meandering and last for few consecutive beats.

Mechanisms underlying the perpetuation of AF are still debated. Multiple wavelets and localized (focal or reentrant) sources are largely accepted to drive AF. These mechanisms are summarized in Figure 2.

#### Multiple wavelets hypothesis

Multiple wavelets were suggested to perpetuate AF in a mathematical model performed by Moe et al. (1964). In this model, multiple waves randomly propagate through the atria, cause wavebreaks, and give birth to new “daughter” wavelets. Theoretically, the number and the stability of these wavelets prevent AF termination. AF would be sustained as long as the number of wavelets exceeds a critical level. The presence of independent wavelets has been demonstrated in more recent studies (Chen et al., 2000; Reumann et al., 2007; De Groot et al., 2016). However, an important question is whether these wavelets are driving AF or they are just passive and result from the breakup of more organized waves remains unanswered. Chen et al. (2000) analyzed the individual wavelets during sustained AF by identifying the phase singularities using optical mapping. In their models, the wavelets existed for less than one rotation in 98% of the cases. In addition, the number of wavelets decreased between the entrance and the exit of the mapping field. These results suggested that wavelets essentially result from the breakup of high frequency organized waves and as such they are not an independent mechanism that maintains of AF. Recording multiple wavelets during firing from the PVs is an example that supports the passive role these wavelets.

#### Localized AF drivers: (Figure 3)

There is no specific definition of AF drivers. Hansen et al. (2016) defined AF drivers as localized sources of fast, repetitive activity from which activation propagates and breaks down into fibrillatory conduction in the rest of the atria. This definition refers to localized activities without specifying the underlying mechanism. The driving role of these activities is demonstrated by ablation slowing or terminating AF.

**Figure 3:**
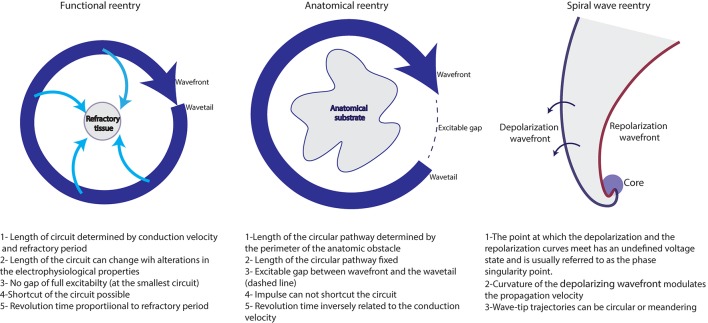
Different reentrant activities maintaining AF (adapted from reference Allessie et al., 1977).

##### Functional reentry

Functional reentry refers to reentrant activity in the absence of underlying substrate and of anatomical obstacles.

*The leading circle concept:* This concept was developed by Allessie et al. (1977). In this theory, centripetal waves moving toward the center maintain this latter refractory. A functional reentry establishes itself in the shortest circuit defined as the shortest distance the impulse travels during the refractory period. The presence of slow conduction velocity (CV) or brief refractoriness (RP) produces a small wavelength (WL) and makes spontaneous termination of AF unlikely. In fact, as WL= CV ^*^ RP, the occurrence of a steady state where the WL is adapted to the circuit length would stabilize the reentry and would perpetuate AF.

*Spiral wave reentry= rotor concept:* Spiral wave reentry or “rotor” is a region of specific reentry where the curved wavefront and wavetail meet each other at a singularity, and where the central tissue is not refractory (Vaquero et al., 2008; Pandit and Jalife, 2013; Nattel et al., 2017).

Evidence for spiral wave reentry was first demonstrated in simulation studies (Winfree, 1973; Goldbeter, 1975; Lechleiter et al., 1991; Pertsov et al., 1993a). Its presence in cardiac tissue was demonstrated using optical mapping by Davidenko et al. (1990). The authors induced sustained reentrant activity by using a single appropriately timed premature electrical stimulus applied perpendicularly to the wake of a propagating quasiplanar wavefront. Reentry pivots at high frequency (5–7Hz) around a relatively small group of cells that show only minimal depolarizations (phaseless region) throughout the cycle.

Mandapati et al. (2000) identified micro-reentrant sources localized in 80% of the cases at the posterior LA, close to the left veins. The authors identified high frequency periodic activity limited to small area (10.4 ± 2.8 mm of core perimeter and 3.8 ± 2.8 mm^2^ area). Using high resolution video imaging, the localized sources correspond to vortex like reentry.

Spatio-temporal characterization of the rotor activities was facilitated thanks to phase transformation. Gray et al. (1998) analyzed phase singularities during fibrillation and demonstrated a spatial and temporal organization that under certain conditions give rise to rotors. Phase represents the different stages within 1 cycle of a signal divided into 360° or 2π radians (Umapathy et al., 2010).

Phase analysis characterization of the recorded signals lacks temporal accuracy, Bray and Wikswo (2002) developed an algorithm capable of establishing proper orbits without the need to specify “Tau.” Using the Hilbert transform, phase singularities could be localized closer to the point of initial formation than was possible previously. This transformation is important for the purposes of singularity tracking and investigating electrodynamic interactions. Phase transformation allows to identify the center of the pivoting rotor as an area of undefined phase and is called phase singularity. This center is surrounded by phases ranging between – π and + π (**Figure 5**).

##### Anatomical reentry

Localized reentries play a key role in the maintenance of AF. Reentry occurs in the presence of unidirectional block and of slow conduction that makes the wavelength shorter than the length of the circuit. Such conditions are commonly encountered in the atria of patients with AF, mainly in the presence of fibrosis.

The role of reentry in maintaining AF was demonstrated by Schuessler et al. (1992). In their model, the authors induced AF using a single extra-stimulus and increasing concentrations of acetylcholine and mapped the right atrial activation. They noted an increase in the number of wavelets that tended to stabilize to small, single, relatively stable reentrant circuit in the absence of anatomical barriers. These reentries are facilitated by the occurrence of lines of functional block at the crista terminalis.

Spach et al. (1988) showed micro-anatomic reentry (within 1–2 mm area) that occur in the presence of non-uniform anisotropic conduction and micro-fibrosis of the pectinate bundles of the right atrium (Spach and Dolber, 1986). Hansen et al. (2015) provided the evidence for intramural reentries that anchor to fibrosis insulated atrial bundles. The authors induced AF in explanted Human hearts and mapped intramural activation of the right atrial wall using high resolution optical mapping. They noted stable reentries that anchor to areas with complex architecture marked by increased transmural fiber angle differences and interstitial fibrosis. The majority of the reentries were mapped from the endocardial surface and discrete ablation terminated AF which confirms their driving role. These micro-reentries were also identified in the left atrium (Zhao et al., 2015) at the junction between the left atrial roof and the posterior wall of the left atrium, at an area with abrupt changes in the myocardial fiber orientation. Ablation of the driver maintain AF can unmask drivers with longer cycle length (Hansen et al., 2015). The presence of several temporally competing drivers and secondary drivers may underlie the absence of acute termination by ablation and should motivate repeated mapping of AF.

AF drivers are more frequently recorded in the left atrium. This is attested by shorter AF CL in the left atrium with a gradient LA-RA, the higher rate of AF termination in the LA. Mansour et al. (2001) analyzed left atrial and right atrial dominant frequencies and identified left to right activation gradient that increased after the ablation of the Bachmann bundle and of the inferoposterior interatrial pathway. This observation supports the higher prevalence of AF drivers in the left atrium with fibrillatory conduction to the right atrium. Hocini et al. (2010) tracked the evolution of AF cycle length at the right and atrial appendages. In 70% of the cases, an increase of both cycle lengths occurred after left atrial ablation. Right atrial drivers were recorded in 20% of the cases.

#### Atrial remodeling

Atrial remodeling includes structural and functional alterations including electric, structural, and autonomic remodeling that promote atrial arrhythmias.

##### Electrical remodeling

AF and rapid arrhythmias alter the expression and/or the function of ion channels in a way that promotes arrhythmias (Allessie et al., 2002; Schotten et al., 2011; Wakili et al., 2011; Heijman et al., 2014; Nattel and Harada, 2014). In fact, rapid atrial rate during AF initiates auto-protective mechanisms to reduce the entry of Ca^2+^ inside the cell (Iwasaki et al., 2011). These mechanisms aim to inactivate the Ca^2+^ currents, downregulate I_CaL_, and enhance the inward rectifier K^+^ current. Subsequently, the action potential duration becomes shorter which increases the atrial vulnerability to atrial arrhythmias and stabilizes the mechanisms sustaining AF (**Figure 5**) (Allessie et al., 2002; Nattel, 2002; Schotten et al., 2011; Wakili et al., 2011). In addition, impaired calcium handling leads to contractile dysfunction and subsequent tachycardia-induced atrial cardiomyopathy (Sun et al., 1998).

##### Structural remodeling

Fibrosis represents the most important structural remodeling that promotes AF.

Fibrosis can be reactive (located at the interstitial space) or reparative (replaces dead myocytes) (Silver et al., 1990; Burstein and Nattel, 2008).

Animal studies identified atrial fibrosis in the presence of hypertension (Kistler et al., 2006; Lau et al., 2010a,b, 2013), heart failure (Li et al., 1999; Shi et al., 2002; Shinagawa et al., 2002; Lau et al., 2011), diabetes (Linz et al., 2016), obesity (Abed et al., 2013),… In humans, AF is more frequent in the presence of external stressors predisposing to fibrosis (Sanders et al., 2003; John et al., 2008; Roberts-Thomson et al., 2009; Medi et al., 2011, 2012; Dimitri et al., 2012; Vlachos et al., 2016; Anter et al., 2017; Karam et al., 2017). In addition, when left untreated, AF promotes the expression of genes that enhance the proliferation of fibroblasts and increase extra-cellular matrix secreting function (Burstein et al., 2007; Guo et al., 2012). This underlies the progression of AF to permanent forms by creating a long-term positive feedback loop (Platonov et al., 2011; Yue et al., 2011); the so called AF begets AF hypothesis (Wijffels et al., 1995; Rostock et al., 2008).

Fibrosis increases the separation of the myocytes within sub-endocardial atrial bundles and between the endocardial and epicardial layers leading to endo-epicardial dissociation (Spach and Dolber, 1986; Verheule et al., 2013; Hansen et al., 2017). It forms barriers to the propagation of the activation wavefronts and isolates atrial myocytes. These obstacles affect the wavefront shape and can induce spiral waves through vortex shedding or by causing localized conduction block in narrow isthmuses (Panfilov and Keener, 1993; Cabo et al., 1996; Starobin et al., 1996). The interaction between the wavefront and the boundaries of the fibrotic area are determinant for the wavefront curvature by influencing the propagation velocities and the refractory periods (Comtois and Vinet, 1999; Sampson and Henriquez, 2002). This stabilizes rotor activity (Morgan et al., 2016; Roney et al., 2016) and anchors them to the scar boundaries (Davidenko et al., 1992; Pertsov et al., 1993b; Morgan et al., 2016).

In addition, the fibrotic pattern affects the velocity of the activation wavefront (Kawara et al., 2001; Comtois and Nattel, 2011). In a mathematical model, Tusscher and Panfilov (2005) demonstrated that an increasing number of small and randomly distributed obstacles decrease the conduction velocity but increase the inducibility of wavebreaks and spiral waves in 2D and 3D excitable media. Kawara et al. (2001) analyzed the wavefront activation in explanted human hearts and identified different conduction curves according to the fibrotic pattern. The zones of dense, patchy fibrosis with long fibrotic strands were associated with prominent activation delay. The conduction curve in this situation was dependent on the fiber direction. Conversely, dense, diffuse fibrosis with short fibrotic strands only marginally affected conduction curves.

In a recent study, Vigmond et al. (2016) demonstrated the possibility to induce percolation in a computer model of fibrotic tissue. Percolation was produced as a result of micro-source-sink mismatch with the fibrotic region. This produced low amplitude and long lasting electrograms. Decreasing the cycle length increased the delay needed for the wavefront to exit the remodeled zones and induced reentrant activities. Additional studies demonstrated the occurrence of reentry near the percolation threshold in heterogeneous cardiac tissue including fibrosis (Alonso and Bar, 2013; Alonso et al., 2016).

In addition to fibrosis, structural atrial remodeling includes atrial fatty infiltration, inflammatory infiltration, necrosis and amyloid deposition (Frustaci et al., 1997; Rocken et al., 2002; Leone et al., 2004; Nguyen et al., 2009; Hatem and Sanders, 2014; Venteclef et al., 2015). The role of the adipose tissue in the pathogenesis of AF is well-demonstrated. Adipose tissue has a paracrine effect through the release of adipokines with pro-fibrotic properties. It also forms barriers to wavefront conduction and favor reentrant circuits (Hatem and Sanders, 2014).

##### Autonomic and neural remodeling

The heart has a rich and complex extrinsic and intrinsic autonomic innervation (Janes et al., 1986; Armour et al., 1997; Armour, 2008). The role of this system in the initiation and maintenance of AF is well-demonstrated (Arora, 2012; Chen et al., 2014) and is supported by the circadian variation in the incidence of AF (Viskin et al., 1999; Mitchell et al., 2003).

Neural remodeling including an increase in the atrial innervation occurs in different clinical situations. Animal studies (Jayachandran et al., 2000; Chang et al., 2001; Arora et al., 2008; Ng et al., 2011) demonstrated an increase in the density of sympathetic and parasympathetic innervation with AF. Gould et al. (2006) collected the atrial appendages in patients with AF undergoing cardiac surgery and demonstrated an increased atrial sympathetic innervation in patients with PsAF.

Neural remodeling also occurs after myocardial infarction (Han et al., 2012; Nguyen et al., 2012; Ajijola et al., 2015) and in the presence of cardiomyopathy (Ajijola et al., 2012) and contributes to the occurrence of AF in these populations. Recent therapeutic strategies aiming to modulate the autonomic tone successfully reduced the AF burden in animal models (Richer et al., 2008; Ogawa et al., 2009; Leiria et al., 2011; Shen et al., 2011) and in humans (Pappone et al., 2004; Scanavacca et al., 2006; Po et al., 2009; Katritsis et al., 2013; Pokushalov et al., 2013).

It is important to note that, in contrast with the electrical remodeling, structural remodeling and fibrosis are not reversible and lead to the perpetuation of AF in more complex forms. Early interventions are of major importance to avoid such progression of the disease.

#### Genetic predisposition

Genetic predisposition plays an important role in the occurrence of AF. It is responsible for familial cases with early onset of AF independently of concomitant cardiovascular conditions (Fox et al., 2004; Lubitz et al., 2010; Oyen et al., 2012). AF incidence also shows racial differences, being less prevalent in Blacks, Hispanics and Asians compared to Whites (Dewland et al., 2013).

Up to one-third of the patients with AF had genetic variants that increase the risk of AF. So far, the genome wide association study (GWAS) and international collaborative metanalysis identified at least 30 gene loci associated to AF (Gudbjartsson et al., 2007; Benjamin et al., 2009; Ellinor et al., 2010, 2012; Sinner et al., 2014; Low et al., 2017; Bapat et al., 2018; Campbell and Wehrens, 2018). Variants located close to the paired-like homeodomain 2 (PITX2) gene on chromosome 4q25 have the highest association to AF (Lubitz et al., 2014; Low et al., 2017). The majority of mutations underlying AF affect genes that encode transcription factors related to cardiopulmonary development, cardiac-expressed ion channels and cell signaling molecules (Roberts and Gollob, 2010, 2014; Ellinor et al., 2012). Genome wide association studies have allowed identification of variants potentially linked to AF (Christophersen et al., 2017; Lee et al., 2017; Low et al., 2017; Nielsen et al., 2018a,b; Roselli et al., 2018). These variants frequently require further classification to confirm or eliminate their pathogenicity. Genetic predisposition may influence the response to AF therapies (Darbar et al., 2007; Parvez et al., 2012; Benjamin Shoemaker et al., 2013; Huang and Darbar, 2016) and can allow specific and based-mechanism therapies (Roberts and Gollob, 2010; Campbell et al., 2013; Faggioni et al., 2014; Darbar, 2016).

## Mapping of AF

Mapping represents a crucial step to understand the mechanisms of AF and improve the results of ablation. However, it is important to note that the spatial resolution of the mapping technique can significantly affect the interpretation of the underlying AF mechanism (Roney et al., 2017).

### Invasive mapping of reentrant and focal activities

Narayan et al. (2012b) used a 64 pole-basket catheter (48 mm diameter, 4 mm electrode spacing; or 60 mm diameter, 5 mm electrode spacing) introduced through a venous femoral access to map AF activities from the right and the left atria. They included 97 patients who underwent 107 consecutive ablation procedures for PAF or PsAF. AF electrograms at 64–128 electrodes are combined with repolarization dynamics acquired using monophasic action potentials (MAP) and conduction dynamics to construct spatiotemporal AF maps (Narayan et al., 2012a). These maps were used to locate the focal impulses (defined as centrifugal activation contours from an origin) and rotors (defined as sequential clockwise or counterclockwise activation contours around a center of rotation emanating outwards to control local AF activation). Rotors and focal impulses were present in 97% cases with sustained AF. The majority of the AF sources were rotors (70%) and predominantly located in the left atrium (76%). In contrast to our experience, no fragmented signals were recorded at the rotors site. The mean AF sources was 2.1 ± 1.0 and was significantly higher in PsAF than PAF and in spontaneous than induced AF. A group of patients underwent ablation targeting Focal Impulse and Rotor Modulation (FIRM guided ablation). Compared to patients undergoing conventional AF ablation, FIRM guided ablation was associated to a higher acute success and a better outcome.

Different systems were developed to invasively map rotational activities (Daoud et al., 2017; Grace et al., 2017; Honarbakhsh et al., 2017). The systems use different approaches and more studies are needed to evaluate their clinical usefulness.

#### Limitations of invasive mapping of reentrant and focal activities

The FIRM approach needs the use of two basket catheters for concomitant bi-atrial analysis. Poor electrode contact and inefficient deployment may significantly alter the recorded signals (Laughner et al., 2016; Oesterlein et al., 2016). In addition, low resolution of mapping the atria may lead to false detections (Roney et al., 2017). Offline analysis is needed which prolongs the duration of the procedure and limits the reproducibility of the results (Benharash et al., 2015; Buch et al., 2016). In addition, there are significant discrepancies between 2D and 3D phase maps where rotors identified using 2-D maps were absent in 3D maps (Pathik et al., 2018).

### Non-invasive mapping

#### Principles of non-invasive mapping

Electrocardiographic mapping (ECGi) is a technology that allows to map the activation of AF from chest recordings in a beat-to-beat manner (Oster et al., 1997; Ramanathan et al., 2004; Cuculich et al., 2010; Frontera et al., 2018a). This technique is particularly useful to map focal sources and non-sustained arrhythmia (Jia et al., 2006; Wang et al., 2011; Shah et al., 2013; Zhang et al., 2013; Cochet et al., 2014).

Recording of the cardiac activity is acquired from the torso using a 252 electrode vest. The cardiac geometry and the position of each electrode is registered using high-resolution cross sectional non-contrast computed tomography. The ECGi algorithm computes epicardial unipolar electrograms from the input geometry and torso potentials by solving the inverse problem (Gulrajani et al., 1988; Rudy and Messinger-Rapport, 1988; Rudy and Oster, 1992; Ramanathan et al., 2003; Rudy, 2013). To avoid the superposition of the QRS, windows with long R-R interval exceeding one second allow to analyze the fibrillatory waves.

Additional algorithms can be applied to acquire different maps. Activation maps are computed using the unipolar electrogram intrinsic deflection (dV/dt) based method. Phase analysis identifies reentrant and focal activities. Reentrant activity is identified as a phase singularity formed at the intersection of depolarization and repolarization isolines (Gray et al., 1998). Focal breakthroughs are shown as activities raising from discrete points and showing a negative pattern of the local electrogram.

#### Results from non-invasive mapping

Cuculich et al. (2010) first used ECGi to map AF in 26 patients. The spatial accuracy for determining different pacing sites was 6 ± 4 mm. The authors identified multiple wavelets (defined as contiguous area of epicardial activation lasting ≥5 ms) as the most common pattern (92% of the patients). Rotor activity was present in 15% of the cases, only in patients with non-paroxysmal AF. The authors defined a complexity index as the sum of the number of wavelets and focal activities and showed an increased complexity with duration of AF.

**Figure 4:**
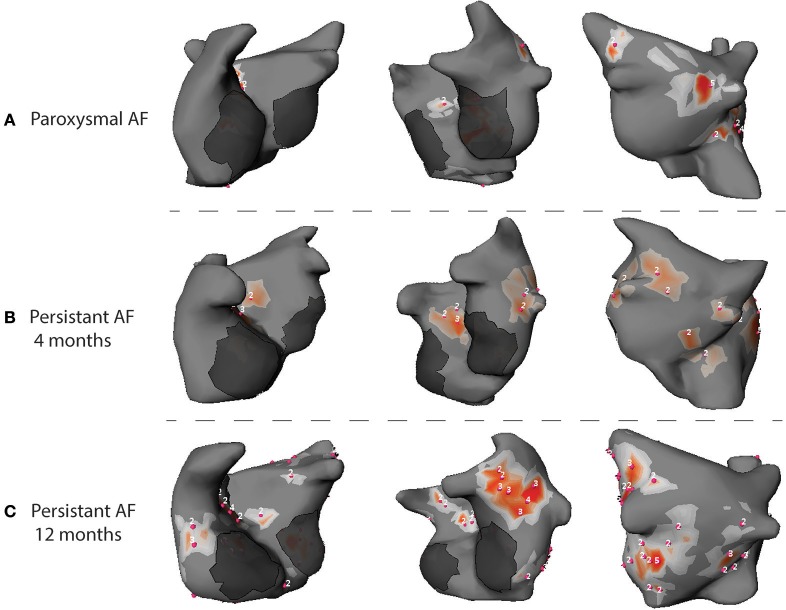
Phase maps acquired during AF in patients with PAF **(A)**, PsAF of 4 months **(B)** and long lasting PsAF >12 months **(C)**. Red spots identify sites of phase singularity.

Data from our laboratory (Haissaguerre et al., 2014; Lim et al., 2017) reported the results of 103 patients with PsAF. The analysis of cumulative windows of 9 ± 1 s of AF were performed using phase mapping. AF was driven by two to three regions of reentrant and focal activities during the first months. Drivers activity spread in more regions and became bi-atrial in PsAF of longer duration (Figure 4). Reentrant drivers were located in the PV antra and surrounding structures, the left atrial appendage and septum in nearly all the cases. Focal breakthroughs rose predominantly from the PV ostia and left and right appendages.

In the AFACART study (Knecht et al., 2017), ECGi was used to guide ablation in 118 patients with PsAF lasting <1 year. Reentrant activities were identified in all patients and were more frequently located around the PVs, at the anterior interatrial groove and the posterior and inferior left atrium. Focal breakthroughs were mapped in 95% of the cases and were more commonly located in the PVs and both appendages.

Metzner et al. (2017) used a noninvasive epicardial and endocardial system (NEEES) and compared the epicardial and the endocardial reentrant activity. The authors acquired phase maps from 6 patients with PsAF. The majority of the epicardial rotor activity was located in two to three anatomical clusters. These results were reproduced using invasive mapping by a multipolar catheter.

The effects of antiarrhythmic drugs were analyzed in a group of 13 patients who underwent ablation for PsAF (Amraoui et al., 2016). ECGi recordings were acquired before and after the infusion of flecainide. Flecainide infusion reduced the number of regions that hosted reentrant activity (7–4 regions, *p* < 0.001). Importantly, AF was terminated to sinus rhythm in 11 cases, by targeting the regions remaining after flecainide infusion in 9/11 cases. This result suggests that anti arrhythmic drugs select more stable and important regions that sustain AF. Similarly, amiodarone was used in patients with structural heart disease and PsAF and allowed to terminate AF using a shorter duration of radiofrequency (Cheniti et al., 2016). In our practice, an antiarrhythmic drug is used before ablation for PsAF order to limit the effects of the electrical remodeling.

#### Limitations of non-invasive mapping

**Figure 5:**
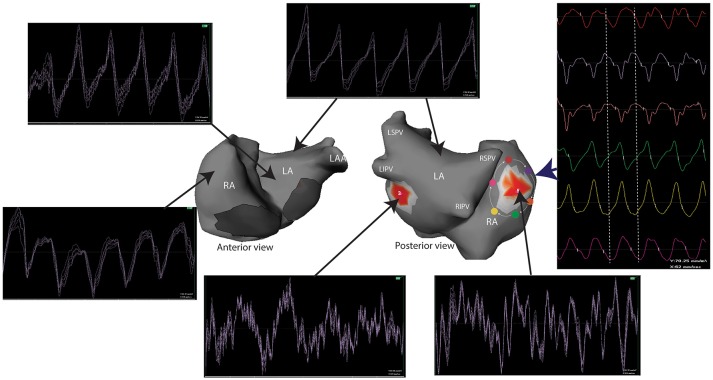
Unipolar signals recorded during a one second window of AF. Electrograms at the site of reentrant activities (red spots) show a complex and turbulent activity while the activity in the remaining atria is homogeneous. A reentry can be identified by analyzing the surrounding electrograms (white arrows) that show a sequential temporal activation. LA, left atrium; LAA, left atrial appendage; LIPV, left inferior pulmonary vein; LSPV, left superior pulmonary vein; RA, right atrium; RIPV, right inferior pulmonary vein; RSPV, right superior pulmonary vein.

Non-invasive mapping has some limitations that should be considered for optimal use. Cardiac signals are attenuated while crossing the thorax, leading to a “blurred vision” by the recording electrode on the torso. Subsequently, the recorded signals by each electrode on the torso represents the average of multiple signals. However, areas with turbulent activity can still be distinguished from areas with more organized activity (Figure 5).

ECGi does not explore intra-mural and endocardial activities. Subsequently, it is unable to discern the mechanisms of focal activities that may be microreentry, epicardial breakthrough of endocardial activity, focal activity (Cuculich et al., 2010). It is also unable to accurately analyze the septal activity where wavefronts from the left and right atria can be projected. In addition, phase transformation can produce phase generated non-rotational singularity points and false rotors (Vijayakumar et al., 2016). In our practice, signals at the reentrant site are manually validated by showing electrograms that cover all the cycle length.

### Invasive mapping of AF

Invasive mapping is limited by the inability to map simultaneously both atria, by contact issues, by mechanical movement of the atrial walls, by the inability to explore intra-mural and epicardial activities. The meandering nature of the sources maintaining AF is an additional major limitation to conventional sequential invasive mapping. Multipolar catheters with small electrodes improved the mapping of AF by acquiring high density maps and reducing the influence of farfield signals.

#### Findings during invasive mapping of AF

Konings et al. (1994) used a spoon shaped electrode containing 244 unipolar electrodes to map right atrial free wall in patients undergoing surgical ablation of accessory pathways. The authors identified 4 major patterns of activation according to the complexity of the atrial activation. Single broad wavefronts propagating uniformly across the right atrium were recorded in 40% of the cases. One or two non-uniformly conducting wavelets were recorded in 32% of the cases. Highly fragmented signals with more than two wavelets and variable direction of propagation were less frequent and recorded in 28% of the cases. The authors correlated the morphology of the signals recorded to the underlying mechanism (Konings et al., 1997). Unipolar signals were different according to the underlying mechanisms, showing single potentials in uniform conduction, short double potentials in areas of collision, long double potentials in areas with conduction block and fragmented potentials in pivoting points and in the presence of slow conduction. No preferential anatomic sites for double or fragmented potentials were found in the right atrium. The authors hypothesized that electrograms spanning the entire cycle length of the AF could identify localized reentries and areas where electrograms displayed fractionation could be pivotal points of these circuits. Fragmented signals with long duration are referred to as complex fractionated atrial electrograms (CFAE) and represented an important target for AF ablation (Nademanee et al., 2004). Different algorithms were developed in order to automatically locate the areas of CFAEs (Scherr et al., 2007; Verma et al., 2008; Seitz et al., 2013; Namino et al., 2015).

Rostock et al. (2006) performed high density endocardial mapping during AF using a 20-pole catheter. The authors identified two patterns of local activation. In the majority of the cases, they recorded nearly simultaneous activation covering only a limited part of the cycle length (≤30% of the AF cycle length). This pattern was correlated to passive activation. More rarely, they recorded complex activation covering more than 75% of the cycle length. These signals were correlated either to local burst activity with activation gradient in the adjacent splines and may be related to localized reentry.

Haïssaguerre et al. (2006) used a 20-pole catheter with 5 radiating splines covering 3.5-cm diameter to map both atria. The authors identified activity spanning 75–100% of the cycle length suggesting a complex localized activity or localized reentry. Ablation targeting these areas significantly prolonged the AF cycle length demonstrating the critical role of these reentries in the maintenance of AF. This result is consistent with the findings of Hansen et al. (2016) identified micro-reentrant activity with average area around 15^*^ 6 mm with 3 mm depth. As such, these reentries may be mapped using high resolution catheters, but only if they are located on the endocardial or epicardial surface.

High density endocardial mapping at the drivers' area identified prolonged fractionated signals. These signals were more frequent in the driving area than in the remaining areas (62 vs. 40%, *p* < 0.001). Most importantly, electrograms recorded on the multispline catheter spanned across a greater part of AF cycle length in the driver regions than elsewhere (71 vs. 47% of the AF cycle length, *P* < 0.001) (Haissaguerre et al., 2014). This suggests a slow conduction or a localized reentry. These electrograms were rarely recorded for more than five consecutive beats indicating an unstable local propagation (Haissaguerre et al., 2016). In addition, these signals may show dynamic changes that are dependent on the local cycle length (Rostock et al., 2006). The instability of the electrograms is suggested by the smoothing of the local activity which shows a turbulent activity. In fact, unipolar signals at the site of reentry identified complex and turbulent activity spanning all the cycle length, while activation in the remaining atria are more homogeneous (Figure 5). In our experience, the persistence of complex activity at driver sites after ablation is associated to the persistence of AF and demonstrates the necessity for further ablation.

#### Limitations of the mapping of fragmented signals

Multiple parameters may affect the accuracy of invasive mapping. The electrode size, the inter-electrode spacing, the proximity to the atrial wall and the duration of the mapping at each site represent the main parameters. Mapping catheters with small electrodes provide a higher sensitivity to near-field signals compared to 4 mm tip catheters (Stinnett-Donnelly et al., 2012; Berte et al., 2015). In addition, fractionation increases as interelectrode spacing increases (Correa De Sa et al., 2011; Lau et al., 2015).

The correlation between the fragmented signals and the underlying mechanism is poor. In fact, complex and fragmented signals may result from artifacts, inappropriate filtering, remote activation related to adjacent structure or overlying structures and alterations in conduction velocities related to wavefront curvature and tissue discontinuities (De Bakker and Wittkampf, 2010). In addition, these fragmented signals are frequently passive. Jadidi et al. (2012) acquired high density atrial maps during sinus rhythm, CS pacing and during AF. The distribution of the fragmented electrograms was different according to the site and the rate of activation. During sinus rhythm and CS pacing, electrogram fragmentation mainly resulted from wavefront collision.

Recent work from our laboratory characterized the mechanism underlying the different types of electrograms using high density mapping (Rhythmia, Boston Scientific) during atrial tachycardia. Frontera et al. (2018b). analyzed electrograms at the sites of slow conduction areas, at the lines of block, at areas of collision and at pivot sites. Areas with slow conduction had a significantly lower amplitude and a long duration. Areas of wavefront collision had a shorter amplitude and a higher voltage. Electrograms at the lines of block were not fragmented, the block lines being defined as areas where the activation completely stopped with the front making a detour around the obstacle, the downstream activation proceeding toward the line of block being in an opposite direction to the upstream one. These characteristics should be assessed during AF.

### Contribution of imaging modalities to AF mapping

Cardiac magnetic resonance (CMR) studies demonstrated a higher proportion of atrial fibrosis in patients with AF compared to healthy patients (Oakes et al., 2009) and in patients with PsAF compared to those with PAF (Oakes et al., 2009; Daccarett et al., 2011).

Oakes et al. (2009) validated a processing protocol to detect atrial fibrosis by using late gadolinium enhancement (LGE) on CMR. This technique was used to characterize the atrial substrate in a group of 81 patients undergoing PV isolation. Fibrosis was present in all cases and its extent predicted the recurrence of AF after PVI. In the DECAAF study (Marrouche et al., 2014), Marrouche et al. demonstrated that an increase of 1% in the proportion of atrial fibrosis was associated to 6% increase in rate of recurrent arrhythmia after catheter ablation. In addition, residual fibrosis on MRI, defined as preablation atrial fibrosis not covered by ablation scar, was associated to the recurrence of AF (Akoum et al., 2015). These studies confirm the key role of fibrosis in the pathogenesis of AF.

Electrograms at the areas with fibrosis were analyzed by Jadidi et al. (2013) in a group of patients undergoing ablation for persistent and long-lasting PsAF. Atrial fibrosis was associated with lower amplitude and a slower and more organized activity. However, complex fractionated atrial electrograms were recorded outside the areas of fibrosis in 90% of the cases.

Haissaguerre et al. (2016) analyzed the presence of fibrosis and its density within each 2.5 mm spherical atrial volume in 13 patients undergoing CMR. The borders of the fibrotic areas hosted the majority of the reentrant activities. In fact, 80% of the reentrant activities were located in areas with a fibrotic density >70%. Conversely, only 10% of the non-driver region harbored such a high fibrotic density.

In a recent study, Cochet et al. (2018) used high resolution LGE-CMR to characterize atrial fibrosis in patients with AF undergoing ECGi guided catheter ablation. The authors characterized LGE density at the reentrant sites. Fibrosis was significantly associated with the number of reentrant regions, to the left atrial volume and to the AF duration. Interestingly, reentrant activities were predominantly clustered at the LGE borders. Moreover, areas with high reentrant activity had a significantly higher local LGE density.

Fibrosis identified by CMR was shown to be an independent factor of AF recurrence after catheter ablation. In a post-analysis of the DECAAF study, Akoum et al. (2015) analyzed LGE CMR 3 months after the ablation in 177 cases. Baseline fibrosis and residual fibrosis were significantly correlated to the need for repeat catheter ablation. Similar results were reported in other studies (Oakes et al., 2009; Malcolme-Lawes et al., 2013; Khurram et al., 2016). Interestingly, CMR studies demonstrated a low rate of complete encirclement of the four pulmonary veins, only in around 7% of the cases (Badger et al., 2010; Akoum et al., 2015). Incomplete PVI is associated to higher recurrence after AF ablation (Peters et al., 2009; Badger et al., 2010). These results raise multiple questions about the efficiency and the durability of lesions caused by ablation.

## Implications for AF ablation

Clinical AF ablation provides clues about the understanding of AF pathophysiology. In Table 1 are presented the results of the main studies and the outcome after percutaneous AF ablation. Unfortunately, there is a significant heterogeneity between the different studies leading to poor reproducibility of the results.

**Table 1:** Summary of the different approaches of percutaneous ablation of AF.

**References**	**Population**	**Ablation technique**	**Acute results/main findings**	**Long term outcome**
**PVI**				
Haïssaguerre et al., 1998	45 PAF	Earliest site of activation of the ectopic beat initiating AF	69 ectopic sites, 94% originating from the PV	62% AF freedom after 8 ± 6months
Chen et al., 1999	79 PAF	Earliest site of activation of the ectopic beat initiating AF	116 ectopic foci, 88.8% originating from the PV	86% AF freedom after 6 ± 2 months, Focal stenosis in 42.4% of the PVs
Haissaguerre et al., 2000	70 PAF	PV isolation (except RIPV) by targeting atrial breakthroughs		73% AF freedom after 4± 5 months, (29 patients had re-ablation session)
Deisenhofer et al., 2003	• 69 PAF • 6 PsAF	Segmental PVI	PVI achieved in 89% of the veins	51% AF freedom after 230 ± 133 days 40% underwent second procedure: 90% due to PV reconnection + 40% extra-PV foci
Arentz et al., 2003	• 37 PAF • 18 PsAF	Segmental PVI	PVI achieved in 99% of the veins	27 pts underwent a second procedure 62% event free after one-year follow-up 70% for PAF, 44% for PsAF
SMART AF Natale et al., 2014	• 172 PAF	• 160 PVI using contact force sensing catheter additional atrial ablation in 50% of the cases		• Atrial arrhythmia freedom after 1-year follow-up ◦ 74% = symptomatic arrhythmia ◦ 69.9% = symptomatic and asymptomatic arrhythmia Contact force within the selected range ≥80% of the time significantly increased the 12 month AF/AT freedom (88% vs. 66%)
STAR-AF study Verma et al., 2015	589 PsAF	67: CPVI, 263: CPVI plus CFAE, 259: CPVI plus linear lesions (roof, mitral isthmus)		CPVI + CFAE and CPVI + lines were not superior to CPVI alone after 18% follow-up (AF freedom = 49,46, 59% respectively, *P* = 0.15)
CHASE-AF trial Vogler et al., 2015	153 PsAF	78 pts PVI alone, 75 full defragmentation	• PVI group: SR achieved with electrical cardioversion • Full defragmentation group: AF termination in 60% (AT = 60%, SR = 40%)	No difference in the AF freedom after 1-year follow-up: 61.4% in the PVI group, vs. 58.3% in the full-defrag group
FIRE AND ICE trial Kuck et al., 2016a	762 PAF	• 378: PVI using cryoablation 384: Segmental PVI using radiofrequency ablation	• Successful PVI • 97.9% in radiofrequency group • 98.9% in the cryoballoon group	• AF and AT freedom without anti-arrhythmic drugs after a mean of 1.5-year follow-up was not different between the two groups: ◦ 65.4% in the cryoballoon group ◦ 64.1% in the radiofrequency group
Alster-Lost-AF Trial Fink et al., 2017	• 69 PsAF 6- 12 months 49 PsAF ≥ 12 months	• 61 pts CPVI-only • 57 pts Substrate-modification group, (CPVI + CFAEs and linear ablation)	• AF termination ◦ CPVI alone = 3% ◦ Substrate modificatio *n* = 19% (*P* = 0.007)	• AF freedom after 1 year follow-up and a single procedure without AAD: ◦ 39% in the CPVI group ◦ 323% in the substrate modification group • AF freedom after 1-year follow-up and a single procedure ± AAD: ◦ 54% in the CPVI group ◦ 57% in the substrate modification group • AF freedom after 1-year follow-up and multiple procedures • 69% in the CPVI group • 86% in the substrate modification group
CASTLE AF trial Marrouche et al., 2018	• 363 pts NYHA II,III,IV with PAF or PsAF, LVEF ≤35% and an ICD • 118 PAF • 245 PsAF • 106 PsAF > 12months	• Ablation = 179 pts vs. medical therapy = 184 • Ablation consisted in PVI plus additional lesions at the discretion of the operator		• Ablation significantly reduced death from any cause and hospitalizations for worsening heart failure ◦ 28.5% after ablation ◦ 44.6%with medical treatment (hazard ratio, 0.62; 95% confidence interval, 0.43 to 0.87; *P* = 0.007)
TIlz et al., 2018	161 PAF	CPVI using EAM and double-Lasso technique	• All PVI were isolated during the index procedure • Up to 5 redo procedures were performed • Recurrence were mainly due to PV reconnections	• 10-year AF freedom ◦ 32.9% after a single procedure ◦ 62.7% after multiple procedures • 6.2% progression to persistent AF after 10 years
**CFAE ABLATION**				
Nademanee et al., 2004	• 57 PAF • 64 PsAF	CFAE ablation	Acute termination without antiarrhythmic drugs • PAF: 86% • PsAF: 62%	• AF freedom after 1 procedure at 1year follow-up = 76% • Overall 91% AF freedom after 1 year follow-up
Oral et al., 2007	100 PsAF	CFAE ablation	Acute termination without antiarrhythmic drug: 16%	• AF freedom after 14 ± 7 months = 33% • Redo ablation = 44% • Overall AF freedom after 13 ±7 months = 57%
Oral et al., 2009	119 long lasting PsAF	• 19 PVI • 50 CFAE ablation • 50 cardioversion	• AF termination by PVI only = 16% • Acute AF termination during CFAE ablatio *n* = 18%	AF freedom after 1 procedure: 36% in the absence of CFAE ablation and 34% after CFAE ablation (P = NS) No benefit of additional CFAE ablation
SELECT-AF study Verma et al., 2014	• 48 PsAF • 28 PAF	• 38 pts: CPVI and all CFAE • 39 pts: CPVI and selective CFAE with continuous electrical activity	• CFAE ablation prolonged AF cycle length and resulted in similar rates of • AF termination (37% vs. 28%; *P* = 0.42)	AF, AFL and AT freedom without anti-arrhythmic drugs after 1-year follow-up significantly lower after selective CFAE ablation (28% vs.50%)
Atienza et al., 2014 RADAR-AF	• PAF = 115, AF induced in 95 pts • PsAF = 117, AF induced in 22pts	• PAF = CPVI or high frequency sources ablation (HFSA) • PSAF = CPVI or CPVI+ HFSA	• AF termination ◦ PAF • CPVI = 38% • HFSA = 58% ◦ PsAF • CPVI = 26% • CPVI + HFSA = 46%	• AF/AT freedom after 1 year-follow-up after a single procedure ◦ PAF • 79% after CPVI • 81% after HFSA • PsAF ◦ 72% after CPVI • 76% after CPVI + HFSA
Faustino et al., 2015	PAF: 150	• 75 PVI alone • 75 PVI + stepwise ablation (CFAEs + linear ablation)	• AF termination and non-inducibility achieved in 100% of the stepwise approach	• AF freedom after a first procedure at 1-year follow up significantly higher in the stepwise group: ◦ 73,3% in the stepwise group ◦ 53.3% after PVI (p < 0.01) • Similar results after a second procedure
Seitz et al., 2017	33 PAF 119 PsAF	• 105 = ablation only regions displaying electrogram dispersion during AF • 47 = PVI and stepwise approach	• Ablation only at dispersion areas terminated AF in 95% of the pts. PVI/stepwise approach terminated AF in 60% of the pts	• AF freedom after a mean of 1.5 procedures per patient procedures after 18 month-follow-up: ◦ 85% = ablation only at regions displaying electrogram dispersion • 59% = PVI/Stepwise approach (*P* < 0.001)
**ROTOR ABLATION AND FIRM ABLATION**				
Cuculich et al., 2010	• PAF: 11 • PsAF ≤12 months: 19 • PsAF >12 months:6	Driver domains identified by ECGi	• Multiple wavelets: most common pattern (92% of the patients) • Rotor activity detected in only 15% of the cases and only in patients with PsAF • AF complexity increased with the AF duration	N/A
Haissaguerre et al., 2014	• PsAF in SR = 26 • PsAF AF ≤12 months = 57 • PsAF > 12 months = 20	Driver domains identified by ECGi	80% AF termination after 28 ± minutes of RF ablation. AF complexity increased with AF duration	85% AF freedom at 12 months in group, no difference compared to the control group
Lim et al., 2017	• PsAF in SR = 32 • PsAF AF ≤12 months = 45 • PsAF > 12 months = 28	Driver domains identified by ECGi	• 70% AF termination, • Increased AF complexity and reduced success rate with the increase of AF duration	NA
Knecht et al., 2017	• PsAF in SR = 32 • PsAF AF ≤12 months = 45	Driver domains identified by ECGi in 8 different centers	64% AF termination after 46 ± 28min RF ablation	• AF freedom after 1-year follow-up was 77% • Of the patients with no AF recurrence, 49% experienced at least one episode of atrial tachycardia (AT) which required either continued AAD therapy, cardioversion, or repeat ablation
Narayan et al., 2012b	• PAF = 31 • PsAF = 76	FIRM guided: 36 Conventional ablation: 71	FIRM guided AF termination in 56% of the cases vs. 9% with conventional ablation	82% in the FIRM guided ablation vs. 45% AF freedom after 9 months
Pappone et al., 2018	PsAF: 81	• Group I: ablation of repetitive-regular activities followed by modified CPVI (mapping group; *n* = 41) • Group II: modified CPVI (control group; *n* = 40)	61% (25/41) AF termination in the mapping- guided ablation vs. 30% (12/40) with conventional strategy (*P* < 0.007)	• AF freedom after 1-year follow-up ◦ 73.2% AF-free recurrence in the mapping group ◦ 50% in the control group (*P* = 0.03)
Honarbakhsh et al., 2017	20 PsAF	• Driver domains identified by basket catheters • Drivers identified using global activation propagation and not phase mapping	• 30 potential drivers: 19 showing rotational activity and 11 focal • 26 drivers were ablated with a predefined response 84% of the cases (AF terminated with 12 and CL showed prolongation ≥30 ms with 10)	N/A
Cochet et al., 2018	PsAF = 41	Driver domains identified using ECGi during AF	• Left atrial (LA)LGE imaging significantly associated with the number of re-entrant regions (R = 0.52; *P* = 0.001) • Clustering of re-entrant activity at LGE borders • Areas with high re-entrant activity showed higher local LGE density as compared with the remaining atrial areas • Failure to achieve AF termination during ablation was associated with higher LA LGE burden, higher number of re-entrant regions and longer AF duration	AF freedom after 11 +/1 2 month-follow-up 25/34 (74%) pts.
**LINEAR ABLATION**				
Jais et al., 2004	PAF = 100	PVI + MI line vs. PVI + CTI line	PVI was achieved in all the pts, MI block was achieved in 92% of the pts	87% AF freedom without anti arrhythmic drugs after MI ablation after 1-year follow-up vs. 69 in the PVI group
Fassini et al., 2005	• PAF = 126 • PsAF = 61	Randomization: 92 PVI vs. 95 PVI + MI line	MI block was achieved in 72% of the pts	AF freedom at 1-year follow-up: PsAF: 74% after MI line vs. 36%, p < 0.01 PAF: 76% after MI line vs. 62%, p < 0.05
Hocini et al., 2005	• PAF = 90	• PVI + roof line • PVI • Ablation of CTI and ostial PV fragmented signals and non PV triggers in all cases	Roof line blocked in 96% of the cases Perimitral flutter inducible in 22% of the cases	87% Af freedom after roof line after 15month-follow-up vs. 69% in the PVI group
Gaita et al., 2008	• PAF = 125 • PsAF and long lasting PsAF = 79	• 67 PVI alone (41 PAF + 26 PsAF) • 137 PVI plus left linear lesions (84 PAF + 53PsAF/Long-standing AF)	• PVI was acutely achieved in all pts. • MI block in 31% of the cases • Roof block in 92% of the cases • CTI block in all patients	• AF freedom after a single procedure at 12-month follow-up, ◦ PVI alone = 46% for PAF ◦ PVI alone = 27% for PsAF ◦ PVI + lines = 57% for PAF ◦ PVI + lines = 45% for PsAF • AF freedom after a single procedure at 3-year follow-up, ◦ PVI alone: 29% for PAF ◦ PVI alone: 19% for PsAF ◦ PVI + lines: 53% for PAF ◦ PVI + lines: 41% for PsAF • After a second procedure (in about 50% of the cases), long term AF freedom without AAD: ◦ PVI: 62% for PAF ◦ PVI: 39% for PsAF ◦ PVI + lines: 85% for PAF ◦ PVI + lines: 75% for PsAF
Mun et al., 2012	• PAF = 156	• 52 = CPVI, • 52 = CPVI+ roof line • 52 = CPVI+ posterior box	• CPVI = 100%, • CPVI +Roof line block = 80.8% • CPVI + posterior box = 59.6%	• AF freedom after 15.6 ± 5 months of follow-up, ◦ 88.5% = CPVI ◦ 78.8% = CPVI + roof line ◦ 80.8% = CPVI + posterior (*P* = 0.44)
Kim et al., 2015	• PAF = 100	• 50 CPVI • 50 CPVI + posterior box lesion and anterior linear ablation	• CPVI + CTI block = 100% • Anterior Line block = 68% • Posterior box isolatio *n* = 60%	• AF freedom after 16.3 ± 4-month follow-up without AAD: ◦ CPVI = 88% ◦ CPVI + posterior box + anterior line = 84%
Kettering et al., 2017	PsAF = 250	• CPVI + roof line • CPVI alone • Additional MI line (6 pts), and right atrial ablation (11 pts)	• Roof blocked in all cases	• AF freedom after 1-year follow-up ◦ 81% after roof line vs. ◦ 74% after PVI (p = NS) • AF freedom after 3-year follow-up ◦ 72% after roof line 63% after PVI, *P* = 0.04
**SUBSTRATE MODIFICATION (FIBROTIC AREAS AND LOW VOLTAGE AREAS)**				
Jadidi et al., 2016	PsAF = 151	• Group 1: 85: PVI + ablation at low-voltage areas (LVA < 0.5 mV in AF) with fractionated activity or rotational activity or discrete rapid local activity • Group 2: 66: PVI (control group)	• AF termination targeting LVAs with specific electrogram patterns = 73% • AF termination sites colocalized within LVA in 80% and at border zones in 20%	• Single- procedural AF-free survival after 13-month follow-up ◦ 69% = group 1 ◦ 47% = group 2 (*P* < 0.001)
Yamaguchi et al., 2016	PsAF = 117	• Group I: 101 = targeting low-voltage areas (<0.5 mV in SR) ◦ Group Ia:39 = PVI + ablation of LVA ◦ Group Ib:62 = PVI only (LVA not identified) • Group II: 16 = LVA non ablated group, only PVI	• Complete low voltage areas elimination in 92% of the cases • Additional linear lesions in 82% of the cases in group Ia	• AF freedom after a single procedure after 18 ±7 months ◦ 72% = No LVA identified ◦ 79% LVA ablation ◦ 38% No LVA ablation
BIFA trial Schreiber et al., 2017	• PAF = 34 • PsAF = 49 • Long lasting PsAF = 9	• 92 PVI + box isolation of fibrotic area (BIFA) (<0.5 mV bipolar signals in sinus rhythm) • 49 PVI (no fibrotic area identified during mapping)	• Different stages of Fibrotic atrial cardiomyopathy (FACM) ◦ 0 = no detectable voltage <1.5 mV ◦ I = very limited severe fibrosis ◦ II = confluence scar fibrotic areas (<0.5 mV) ◦ III = pronounced ≥ 2 scar fibrotic areas (<0.5 mV) • IV = diffuse fibrosis (“strawberry”)	• AF freedom after 16 ± 8 months ◦ Single procedure = 69% ◦ Multiple procedures = 83% • The extent of fibrosis significantly associated to AF recurrence
STABLE SR Yang et al., 2017	PsAF = 229 pts	• STABLE-SR group: 114 = CPVI + CTI + ablation-homogenization of areas with low-voltage (LVZ 0.1–0.4 mV in SR) and complex electrograms • Stepwise group:115 = CPVI + linear lesions + CFAEs	• AF termination in STABLER-SR group = 12.3% • AF termination in stepwise group = 32.5%	AF-free survival after 18-month follow-up STABLE-SR group: 74% Stepwise group: 71.5% (*P* = 0.325)

*AF, atrial fibrillation; AFL, atrial flutter; AT, atrial tachycardia; CTI, cavo-tricuspid isthmus; EAM, electro-anatomical mapping; LVEF, left ventricular ejection fraction; CPVI, circumferential pulmonary veins isolation; MI, mitral isthmus; PAF, paroxysmal AF; PsAF, persistent AF; PVI, pulmonary veins isolation*.

Catheter ablation is superior to antiarrhythmic drugs in preventing AF recurrence (Hazard ratio = 0.53) as reported in the CABANA trial. However, the best strategy is still to be identified.

Despite the different techniques, CPVI remains the cornerstone of the treatment of AF, regardless of the AF form and of the AF duration (Voskoboinik et al., 2017). No strategy consistently demonstrated superiority to CPVI in preventing long term recurrences of atrial arrhythmias.

It is notable that higher rates of success are reported after multiple procedures (Ganesan et al., 2013). This raises questions about the identification of the mechanisms underlying AF and the efficiency and the durability of the lesions created during index procedures.

### Paroxysmal AF

PV isolation (PVI) is the most widely used technique to treat PAF. The initial strategy targeted the earliest activation site by performing a focal and discrete ablation (Haïssaguerre et al., 1998). However, this technique was associated to high rates of pulmonary vein stenosis (Rostamian et al., 2014). Later, wide area circumferential ablation that disconnects the PV two by two became the strategy of choice. This technique is thought to have better results (Lo et al., 2007) by targeting the trigger sources and the ostial drivers and also by autonomic denervation (Redfearn et al., 2007).

PVI is associated with high rates of freedom from AF recurrence. The freedom rate from AF recurrence varies between 60 and 79% (Katritsis et al., 2008; Takigawa et al., 2015a; Straube et al., 2016; Kis et al., 2017), 60 and 72% at 3 years (Vogt et al., 2013; Takigawa et al., 2015a; Takarada et al., 2017) and decreases to 53–68% at 5 years (Neumann et al., 2013; Takigawa et al., 2015a, 2017; Kis et al., 2017). The long term freedom from AF may reach 77% (Vogt et al., 2013) after redo PVI. AF recurrence may be related to non PV triggers in one half of cases (Takigawa et al., 2015b) and to the reconnection of the PVs in the remaining cases.

Substrate modification in addition to PVI was tested by Di Biase et al. (2009) who randomly assigned 103 consecutive patients undergoing PAF to PVI alone (*n* = 35), PVI followed by CFAE ablation (*n* = 34) or CFAE ablation alone (*n* = 34). There was no difference in terms of success rate between PVI alone and PVI followed by CFAE ablation. However, CFAE ablation alone was associated with the highest rates of AF recurrence after 1-year follow-up. Similar results were reported in subsequent studies (Deisenhofer et al., 2009; Chen et al., 2011; Hayward et al., 2011). In addition, techniques aiming at incomplete PVI (Kuck et al., 2016b) or not isolating PV (Mikhaylov et al., 2011) were associated to worse results.

### CFAE ablation

Targeting the complex fractionated signals was commonly used as a strategy to ablate PsAF forms. Originally in 2004; Nademanee et al. (2004) included 121 patients with paroxysmal AF (57 patients) or chronic AF (64 patients) and performed ablation by targeting fragmented electrograms without additional PVI. They reported a high rate of acute success by targeting areas of CFAE (without PVI) with 95% of AF termination by ablation (associated to ibutilide in 28% of the cases) and 76% of AF freedom at 1-year follow-up after one procedure. However, this result was not reproduced by Oral et al. (2007) who included 100 patients with chronic AF where they ablated CFAEs in the left atrium and the coronary sinus without performing PVI. Only 33% of the patients were free from AF or AT recurrence after a follow-up of 14 ± 7 months after one procedure. A second procedure was performed in 44% of the patients, pulmonary vein tachycardia originating from the targeted veins sustained atrial tachycardia in all cases.

Oral et al. (2009) performed a randomized study and included 119 consecutive patients with long-lasting PsAF. All patients underwent PVI that allowed termination to sinus rhythm in 19 (16%) of the cases. In the remaining 100 cases, patients were equally assessed to either electrical cardioversion or ablation of the CFAE in the left atrium or the coronary sinus. After 10 ± 3-month follow-up, there was no difference in the rate of sinus rhythm without anti-arrhythmic drugs between the 3 groups.

In the STAR AF II study, Verma et al. (2015) performed a prospective randomized multicenter study that included 589 patients with PsAF. Ablation consisted in PVI alone in 67 patients, PVI and ablation of complex fractionated electrograms (263 patients) or PVI and additional linear ablation (259 patients). Acute termination of AF during ablation was significantly higher in patients undergoing PVI and complex electrograms ablation or PVI and linear ablation than patients undergoing PVI alone. However, the freedom from AF was not different between the three groups.

### AF drivers' ablation

In a recent meta-analysis, Parameswaran et al. (2018) analyzed the outcome after rotor ablation in 11 studies with a total of 556 patients undergoing FIRM ablation for paroxysmal AF (*n* = 166) or PsAF (*n* = 390). Pooled single-procedure freedom from AF was around 37.8% PAF and 59.2% for PsAF after a mean follow-up of 1 year. Heterogeneity between the different studies was significantly high.

In the AFACART study (Knecht et al., 2017), non-invasive mapping was used to guide the ablation for 118 patients with PsAF lasting <1 year. Ablation targeted the drivers identified by the system, followed by PV isolation and linear ablation when AF could not be terminated. Ablation targeting the drivers' sites terminated AF in 64% of the cases after a mean radiofrequency ablation duration of 46 ± 28 min. AF termination rate increased to 72% when additional PVI and atrial linear ablation were performed. Extra-PV sources played a key role in the maintenance of PsAF and their ablation is associated with the termination of AF in the majority of the cases and arrhythmia freedom up to 77% at a 1-year follow-up.

### Surgical treatment for AF

Surgical treatment for AF was first described by Dr Cox (Cox, 1991). The surgery consisted in linear incisions of the atrial walls that aimed to interrupt the multiple wavelets and reentrant circuits and subsequently direct atrial activation through a maze-like system involving both atria. Different surgical techniques were developed later (Fragakis et al., 2012; Xu et al., 2016), all associated to high rates of arrhythmia free outcome (Prasad et al., 2003; Ballaux et al., 2006; Barnett and Ad, 2006; Weimar et al., 2012; Gillinov et al., 2015).

Hybrid approach (Tahir et al., 2018) overcomes the limitations of the catheter based ablation and of the surgical ablation. Epicardial thoracoscopic ablation followed by endocardial ablation is associated to high rates of long term freedom from AF recurrence exceeding 70% in patients with paroxysmal or PsAF (Krul et al., 2011; Pison et al., 2012, 2014; La Meir et al., 2013; Kurfirst et al., 2014; Bulava et al., 2015).

## Conclusion

Mechanisms underlying AF are complex and remain incompletely understood despite extensive research. Translating AF mechanisms described in basic science to the clinical practice remains challenging. In contrast with PAF, therapeutic interventions for PsAF are still inadequate, mainly limited by the identification of the sources maintaining AF. PsAF is driven by focal and reentrant activity which are initially clustered in a relatively limited atrial surface. These drivers disseminate everywhere because of the atrial remodeling which increases the complexity of AF. Accurate mapping techniques that consider the spatio-temporal variation of AF are essential to identify these sources.

## Author contributions

All authors listed have made a substantial, direct and intellectual contribution to the work, and approved it for publication.

### Conflict of interest statement

The authors declare that the research was conducted in the absence of any commercial or financial relationships that could be construed as a potential conflict of interest.
